# *De novo* whole-genome assembly of the critically endangered southern muriqui (*Brachyteles arachnoides*)

**DOI:** 10.1093/g3journal/jkaf034

**Published:** 2025-02-17

**Authors:** Christopher Faulk, Carrie Walls, Brandie Nelson, Paloma R Arakaki, Irys H L Gonzalez, Nancy Banevicius, Rodrigo H F Teixeira, Marina A Medeiros, Gessiane P Silva, Mauricio Talebi, Wilson C J Chung, Rafaela S C Takeshita

**Affiliations:** Department of Animal Science, University of Minnesota, Minneapolis, MN 55108, United States; Department of Animal Science, University of Minnesota, Minneapolis, MN 55108, United States; Department of Biological Sciences, Kent State University, Kent, OH 44242, United States; Coordenadoria de Fauna Silvestre, Secretaria de Meio Ambiente, Infraestrutura e Logística, São Paulo, SP 04301-905, Brazil; Centro de Ciências da Natureza, Programa de Pós-Graduação em Conservação da Fauna, Universidade Federal de São Carlos, Buri, SP 18290-000, Brazil; Coordenadoria de Fauna Silvestre, Secretaria de Meio Ambiente, Infraestrutura e Logística, São Paulo, SP 04301-905, Brazil; Departamento de Pesquisa e Conservação da Fauna, Zoológico Municipal de Curitiba, Curitiba, PR 80020-290, Brazil; Departamento de Veterinária, Parque Zoológico Municipal Quinzinho de Barros, Sorocaba, SP 18020-286, Brazil; Hospital Veterinário, Universidade de Sorocaba, Sorocaba, SP 18023-000, Brazil; Programa de Pós-Graduação em Animais Silvestres, Universidade Estadual Paulista, Botucatu, SP 18618-681, Brazil; Departamento de Veterinária, Parque Zoológico Municipal Quinzinho de Barros, Sorocaba, SP 18020-286, Brazil; Instituto de Biodiversidades e Florestas, Universidade Federal do Oeste do Pará, PA, Santarém, PA 68035-110, Brazil; Departamento de Ciências Ambientais, Programa de Pós-Graduação Análise Ambiental Integrada, Universidade Federal de São Paulo, Diadema, SP 09913-030, Brazil; Department of Biological Sciences, Kent State University, Kent, OH 44242, United States; School of Biomedical Sciences, Kent State University, Kent, OH 44242, United States; School of Biomedical Sciences, Kent State University, Kent, OH 44242, United States; Department of Anthropology, Kent State University, Kent, OH 44242, United States

**Keywords:** southern muriqui, nanopore, whole-genome sequencing, mitogenome, methylation

## Abstract

The southern muriqui (*Brachyteles arachnoides*) is one of the 2 species of muriquis (genus *Brachyteles*), the largest body-sized nonhuman primate from the Neotropics. Deforestation and illegal hunting have led to a continuing decline in the muriqui population, leading to their current classification as critically endangered. The lack of a reference genome for the genus *Brachyteles* prevents scientists from taking full advantage of genomic tools to improve their conservation status. This study reports the first whole-genome assemblies of the genus *Brachyteles*, using DNA from 2 zoo-housed southern muriqui females. We performed sequencing with Oxford Nanopore Technologies’ PromethION 2 Solo using a native DNA library preparation to preserve DNA modifications. We used Flye to assemble genomes for each individual. The best final assembly was 2.6 Gb, in 319 contigs, with an N50 of 58.8 Mb and an L50 of 17. BUSCO completeness score for this assembly was 99.5%. The assembly of the second individual had similar quality, with a length of 2.6 Gb, 759 contigs, an N50 of 47.9 Mb, an L50 of 18, and a BUSCO completeness score of 99.04%. Both assemblies had <1% duplicates, missing, or fragments. Gene model mapper detected 24,353 protein-coding genes, and repetitive elements accounted for 46% of the genome. We also reported the mitogenome, which had 16,562 bp over 37 genes, and global methylation of CpG sites, which revealed a mean of 80% methylation. Our study provides a high-quality reference genome assembly for the southern muriqui, expanding the tools that can be used to aid in their conservation efforts.

## Teaser

The southern muriqui (*Brachyteles arachnoides*) is one of the largest nonhuman primate from the Neotropics, but deforestation and illegal hunting have led to a substantial and continuing decline in the muriqui population. Genomic tools have been critical to design strategies to promote conservation of endangered species. Here, Faulk *et al*. report the first whole-genome assembly of the southern muriqui. This high-quality assembly will serve as reference genome for the species, and will aid in conservation efforts for this critically endangered primate species.

## Introduction

Muriquis (*Brachyteles*) are the largest body-sized primates native to the American continent, known by local populations as “hippie monkeys” due to their relaxed intergroup relationships. While *Brachyteles* was once considered to be a monotypic genus, there is current behavioral, geographical, morphological, and molecular evidence of 2 separate species: northern (*Brachyteles hypoxanthus*) and southern (*Brachyteles arachnoides*) muriquis (Sa *et al.* 1990; Leigh and Jungers 1994; Chaves *et al.* 2019). They are unique among nonhuman primates for their egalitarian social system and reduced canine dimorphism when compared with their sister clade *Ateles* or *Lagothrix*, which makes them excellent models for studies on evolution of social behavior. However, deforestation and illegal hunting have contributed to a decline of ∼80% of their population over 3 generations and a 50% probability of extinction in 50 years (Talebi *et al.* 2021). This alarming situation places muriquis as critically endangered by the IUCN Red List, with only a few thousand individuals remaining in the wild across the genus (de Melo *et al.* 2021; Talebi *et al.* 2021).

**Fig. 1.**
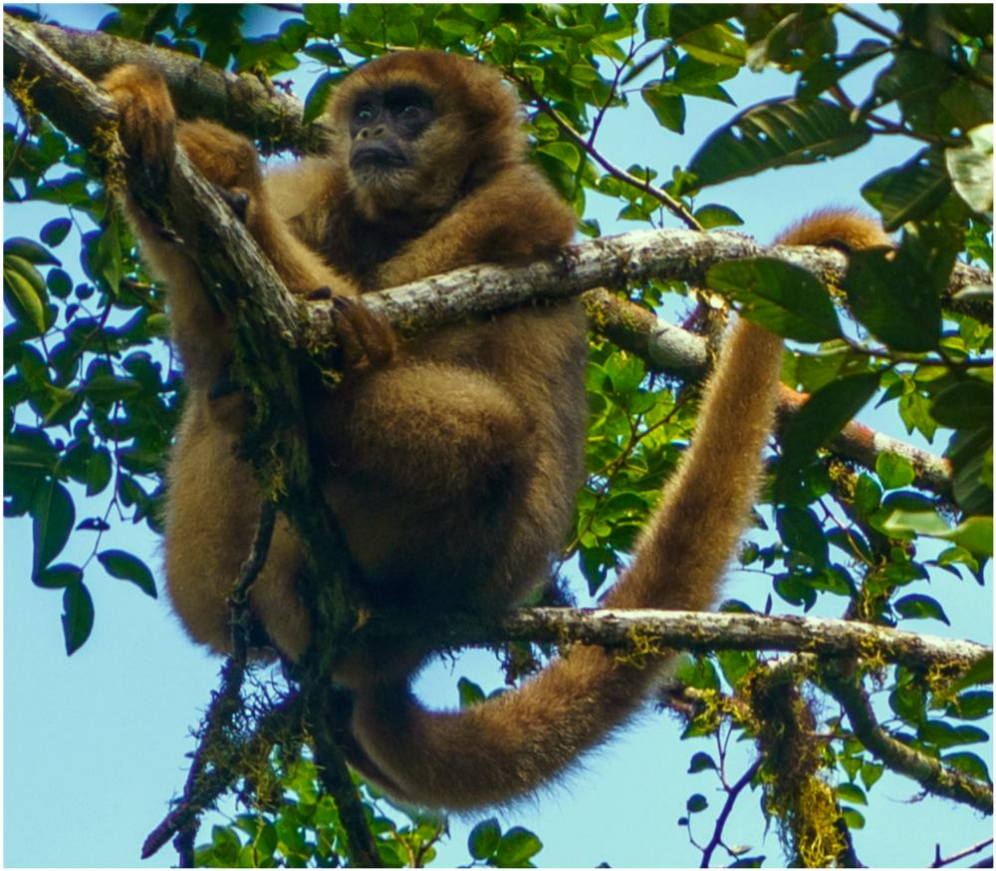
Southern muriqui (*B. arachnoides*) from Parque Estadual Carlos Botelho, São Miguel Arcanjo, SP, Brazil (photo by R Takeshita).

One study analyzed 35 wild muriqui populations (northern and southern) and identified that the southern muriquis from *Parque Estadual Carlos Botelho,* São Miguel Arcanjo, São Paulo (Fig. 1), have the highest level of genetic diversity among all assessed populations (Strier *et al.* 2017). This places them at the highest priority for conservation action plans due to its greatest probability of population persistence. With the advance of genomic tools and the assembly of complete genomes, scientists can obtain genetic data to aid in conservation of endangered species (Supple and Shapiro 2018; Hogg and Belov 2022). For instance, reference genomes have been useful for identification of microsatellites to investigate inbreeding (Zhang *et al.* 2020; Foster *et al.* 2021; Hasselgren *et al.* 2021), mate choice (Hou *et al.* 2018), to detect pathogen-resistant alleles (Kosch *et al.* 2017; Silver *et al.* 2021; Kosch *et al.* 2022), and to identify individuals for long-term monitoring (Ruiz-González *et al.* 2013; Hou *et al.* 2018; Marshall *et al.* 2022). Genomic analyses can be widely employed in nonhuman primate populations to determine genetic diversity, historical events, and parental relationships and to assess population structure and their adaptive potential following reintroduction (Wyner *et al.* 1999; Strier *et al.* 2011; Farias *et al.* 2015; Oklander *et al.* 2021). Yet, there are no complete reference genomes available for either species of muriqui.

The assembly of high-quality genomes has been enhanced by third-generation technologies. In contrast to previous techniques, third-generation sequencing can generate long sequence reads, which makes genome assembly easier and faster, and it increases the accuracy in detecting regions of the genome that are difficult to map, such as short tandem repeats. One of these technologies, developed by Oxford Nanopore Technologies (ONT), is based on electrically charged membranes covered in nanopores, with motor proteins that unzip the double helix into single strands. As each fragmented DNA strand passes through the nanopores via electrophoresis, the electrical signals are used to identify nucleotides that will be converted into sequences (Delahaye and Nicolas 2021; Wang *et al.* 2021). Another advantage of ONT is that it simultaneously detects base modifications, including 5′-methylcytosine (5mC), 5′-hydroxymethylcytosine (5hmC), and N6-methyldeoxyadenosine (6 mA) (Liu *et al.* 2019; Xu and Seki 2020). This enables scientists to identify methylation patterns over the entire genome, which can provide valuable information about epigenetic changes due to the environment [reviewed by Faulk and Dolinoy (2011)]. For example, DNA methylation data can determine long-lasting impacts in an individual due to early-life stressors (Laubach *et al.* 2021) and can monitor how social and environmental conditions affect the epigenome of populations (Faulk *et al.* 2013; Laubach *et al.* 2019; Perera *et al.* 2020), which can have direct implications to their physiology and lifespan (Faulk *et al.* 2014; McLain and Faulk 2018; Soto-Palma *et al.* 2022).

The main objective of this study was to assemble a reference genome for the southern muriqui. In addition, we report the mitogenome and global DNA methylation pattern in 2 zoo-housed individuals using ONT sequencing.

## Material and methods

### Animal subjects

Whole blood samples from 2 female southern muriquis (ages 2 and 4 years) were obtained opportunistically during their routine physical examination from the Passeio Público Municipal de Curitiba, State of Paraná, Brazil. One female (Serena) was born at the Curitiba Zoo (State of Paraná, Brazil), and the other female (Monalisa) was rescued from illegal trade at State of Rio Grande do Sul, Brazil. Immediately upon collection, samples were transferred to DNA/RNA Shield Blood Collection Tubes (Zymo Research) and stored at room temperature for up to 2 weeks until they were transferred to a freezer (−20°C).

The study was considered exempt from Kent State University IACUC review and oversight as no antemortem procedures were on animals for the purpose of this project and all samples were opportunistically obtained. This project was registered in the Brazilian National System of Management of Genetic Heritage and Associated Traditional Knowledge (SISGEN no. A83A517).

### DNA extraction and sequencing

DNA was extracted using a MagAttract HMW DNA kit (cat no. 67563 Qiagen, Inc.) according to the manufacturer's instructions. In-country sequencing was performed on a PromethION 2 Solo instrument (ONT, Oxford, UK) using PromethION R10.4.1 flow cells at the Biotechnology and Clinical Diagnostics Laboratory at the Secretariat of Environment, Infrastructure and Logistics—SEMIL (São Paulo, Brazil). Two libraries were created for each animal sample using the LSK-114 ligation sequencing kit. For the first library, 3 μg of DNA in 100 μL elution buffer was sheared by passage through a 28-gauge needle 30 times, library prepped, and then split into 3 aliquots (32 μL). For the second library, DNA was left unsheared, eluted, and prepped as a single 32-μL library. The first aliquot of the unsheared library was loaded onto the flow cell and run for 24 h, after which the flow cells were washed using the manufacturer's wash kit (ONT, Oxford, UK). Two of the sheared library aliquots were sequenced on days 2 and 3, respectively. Data were collected using 5 kHz MinKNOW version 24.06.8 (ONT, Oxford, UK). Additional sequence data were obtained by creating barcoded libraries using the SQK-NBD114.24 native barcoding gDNA ligation sequencing kit. 500 ng of unsheared DNA for 3 individuals was barcoded, and the library was split into two 32 μL aliquots. The aliquots were run for 24 h each with a flow cell wash in between.

### Base calling

Raw data from all runs were base called post hoc using Dorado v0.8.1 (https://github.com/nanoporetech/dorado) with model dna_r10.4.1_e8.2_400bps_sup@v5.0.0. Base modifications with 5mC and 5hmC were called simultaneously using the Dorado flag-modified-bases 5mC_5hmC. Overall read quality was assessed using Samtools; reads were filtered with quality scores >10. Dorado correct was used to correct reads >5 kb prior to assembly.

### Genome assembly

The obtained sequences were de novo assembled to obtain the southern muriqui genome using Hifiasm v0.16.0 and Flye v2.9.5 (Kolmogorov *et al.* 2019). The FCS-adapter tool from the NCBI Foreign Contamination Screening program suite was used to detect and remove adapter and vector contamination (https://github.com/ncbi/fcs). Haplotigs and contig overlaps were removed using Purge Dups v1.2.6 (https://github.com/dfguan/purge_dups). The resulting draft assembly was scaffolded using NTlink v1.3.10 with gap filling resulting in a more contiguous contig-level assembly (Coombe *et al.* 2021).

### QC and BUSCO

The quality of all draft assemblies was evaluated by detecting Benchmarking Universal Single-Copy Orthologs (BUSCOs) within the lineage (Manni *et al.* 2021). Compleasm was used to calculate BUSCO scores, a faster and more accurate BUSCO implementation (Huang and Li 2023). Compleasm single and duplicate BUSCO counts were combined to provide a direct comparison to the standard BUSCO program's “complete” value.

### Align reads to final genomes

Dorado aligner was used to map the full read set back to the final assembly to create the diploid genome and to calculate DNA methylation per CpG site.

### Diploid genome

HapDup v 0.12 (https://github.com/KolmogorovLab/hapdup) with singularity was used to convert a haploid long read assembly into a diploid assembly.

### Gene annotation

Homology-based gene prediction was performed with Gene Model Mapper v1.8 (http://www.jstacs.de/index.php/GeMoMa) (Keilwagen *et al.* 2019) using human transcripts as the reference. To assess gene prediction accuracy and completeness, BUSCO was set in protein mode with Compleasm.

### Mitochondrial assembly

The mitogenome was extracted from the *B. arachnoides* assembly using MitoHiFi v3.2.2 (Allio *et al.* 2020; Uliano-Silva *et al.* 2023), which identifies mitogenome contigs by comparison to known mitogenomes from related species; in this case, we used the *Ateles geoffroyi* mitogenome (OM328927). MitoHiFi was also used to circularize and annotate the putative mitogenome contig. Multiple alignment programs for amino acid or nucleotide sequences v7.526 (Katoh *et al.* 2002) and iqtree v2.3.6 (Minh *et al.* 2020) were used to generate mitochondrial phylogeny.

### Repeats

Repetitive sequences were identified with RepeatMasker v4.1.7 (https://www.repeatmasker.org) with the complete Dfam library v3.8 (https://www.dfam.org/home) as described previously (Flynn *et al.* 2020; Storer *et al.* 2021). We first attempted to characterize repeats using a novel repeat detection pipeline; however, when we compared our RepeatModeler2 de novo library masked genome vs. the publicly available repeats in the Dfam library, we found that the Dfam database masked a significantly higher percentage of the genome.

### Methylation

Global 5mC and 5hmC at cytosine–guanine CpGs were determined using modified base information stored in the initial base calling output files. The mapped modBAMs were converted to bedMethyl format using Modkit v0.4.1 (https://github.com/nanoporetech/modkit). Global 5mC and 5hmC percentages were calculated using AWK (https://www.gnu.org/software/gawk/manual/gawk.html#Manual-History).

## Results and discussion

Nanopore sequencing yielded a total of 78 and 95 Gb bases from each of the 2 muriqui samples. Estimating the muriqui genome size at 2.6 Gb based on closely related species, these data amount to a coverage of 29.15× and 36.6×, respectively. The read N50s were 25,623 and 13,251 bp, and the quality for each averaged Q20.25 and Q20.6, respectively.

We drafted the initial assemblies using the uncorrected data to compare the performance of Flye and Hifiasm based on the number of contigs and assembly N50 and L50. Flye is based on an algorithm that generates random paths using repeat graphs to reveal genomic repeat structure and align reads (Kolmogorov *et al.* 2019). Hifiasm is based on an algorithm that preserves the continuity of all haplotypes for the purpose of phasing the genome (Cheng *et al.* 2021). The Flye assembly for Monalisa resulted in a 2.6 Gb genome with 1,443 contigs, an N50 of 22.3 Mb, and an L50 of 33. Similarly, Flye assembly for Serena revealed a 2.7-Gb genome draft with 2,013 contigs, an N50 of 18.9 Mb, and an L50 of 41. Overall, Flye outperformed Hifiasm, with longer N50s and lower L50s (Table 1), and therefore, Flye was used for further analyses. This result was consistent with a previous study on the brown-headed spider monkey (*Ateles fusciceps fusciceps*), which reported that the Flye assembly was ∼10 times superior to SMARTdenovo, an Overlap-Layout-Consensus algorithm (Pozo *et al.* 2024).

**Table 1.** Comparative statistics for 2 genome draft assemblies of 2 *B. arachnoides* individuals.

Individual	Assembly method	Length (Gb)	# Contigs	N50 (Mb)	L50
Monalisa	Hifiasm	2,799,423,048	2,787	6.4	108
Monalisa	Flye	2,624,517,278	1,443	22.3	33
Serena	Hifiasm	2,995,493,499	3,666	3.2	211
Serena	Flye	2,691,704,252	2,013	18.9	41

To create the baseline assemblies for the final genome build, we generated new draft assemblies using Dorado-corrected data. The Dorado base caller implements a haplotype-aware algorithm to reduce read errors by comparison of *κ*-mers. These assemblies were then polished, purged of haplotigs, and scaffolded, which improved the final assembly by removing 1,254 contigs and 12 Mb of data from Monalisa and 1,124 contigs and 17.5 Mb from Serena from the initial draft (compare Tables 1 vs. 2). Gaps were filled using ntLink, a scaffolding program with gap filling. After scaffolding, any contigs with unfilled gaps were split and the N's removed, creating a more contiguous contig-level consensus. The final assemblies had N50s of 58.8 Mb and 47.9 Mb and L50s of 17 and 18 for the 2 samples. This result indicates an outstanding genome. Compared with other genome assemblies of closely related species, the genome was assembled into fewer longer length contigs, representing larger sections of chromosomes (Table 2).

**Table 2.** Final genome assembly of 2 *B. arachnoides* following polishing and haplotig purging compared with other Atelids.

Species	Length (Gb)	# Contigs	N50 (Mb)	L50	GC (%)	BUSCO completeness (%)	Reference
*B. arachnoides* (Monalisa)	2.6	319	58.8	17	40.8	99.51	This study
*B. arachnoides* (Serena)	2.6	759	47.9	18	40.9	99.04	This study
*A. hybridus*	2.6	1,315	50.5	16	40.5	99.19	Alioto *et al.* (2021)
*A. geoffroy*	2.7	2,723	29.2	28	41	98.98	Shao *et al.* (2022)
*A. fusciceps*	3.2	3,711	9.8	100	40.5	96.19	Pozo *et al.* (2024)
*L. lagothricha*	2.6	82,380	0.07	10,830	41	75.63	Kuderna *et al.* (2023)
*A. paniscus*	2.6	99,037	58.6	13,029	41	73.02	Kuderna *et al.* (2023)
*A. marginatus*	2.7	132,787	53.9	14,479	41	71.94	Kuderna *et al.* (2023)
*A. belzebuth*	2.6	111,455	51.9	14,646	41	70.96	Kuderna *et al.* (2023)
*A. chamek*	2.6	111,292	0.05	14,750	41	71.09	Kuderna *et al.* (2023)
*A. palliata*	3	1,152,695	51.3	15,704	41	76.16	Johnson *et al.* (2019)

Following, we produced a diploid assembly using the final Flye assemblies. For this purpose, HapDup was used to split each genome into 2 parental haplotypes (Table 2). The BUSCO completeness score of the assembled genome and single and duplicate rates per haplotype are detailed in Table 3. This is of particular importance as haplotype-resolved assemblies can allow insights into genetic diversity estimates. For comparison, we also included the detailed BUSCO scores of the brown spider monkey genome, which was sequenced using ONT's MinION (Pozo *et al.* 2024). Our assemblies for Monalisa and Serena achieved excellent BUSCO scores, indicating that our sequencing and assembly procedure has successfully reconstructed a reliable and complete representation of the full set of genes in the *B. arachnoides* genome with little-to-no duplication, fragmentation, and high contiguity. When compared with other genome assemblies of species within the family Atelidae, our genome assembly from Monalisa had the highest BUSCO completeness score (Table 2).

**Table 3.** Comparison of BUSCO scores between our 2 *B. arachnoides* individuals and that for *Ateles fusciceps fusciceps* (Pozo *et al.* 2024).

BUSCO	*B. arachnoides* (Monalisa)	*B. arachnoides* (Serena)	*A. fusciceps*
Complete	99.51%	99.04%	99.19%
Single	98.85%	98.31%	98.09%
Duplicate	0.66%	0.73%	1.10%
Fragment	0.15%	0.22%	0.15%
Incomplete	0.00%	0.00%	0.00%
Missing	0.33%	0.75%	0.66%
Number	13780	13780	13780

### Gene annotation

Gene model mapper detected 24,353 protein-coding genes using the human T2T genome as a reference, which was similar to that of humans and other primates (Nurk *et al.* 2022; Guigó 2023). The protein BUSCO score was 87.13%.

### Repeats

The number of total interspersed elements (Table 4) detected by RepeatMasker using the Dfam library accounted for 46% of the assembled genome, which is in line with other primate species, such as *Callithrix jacchus*, *Gorilla gorilla*, *Pan troglodytes* (Ahmad *et al.* 2020), and humans (Liehr 2021). As a comparison, we also attempted de novo identification of repeats and found a lower percentage of the genome was masked with this method, with only 38% of the genome marked as interspersed repeats.

**Table 4.** Repetitive DNA content RepeatMasker.

	Number of elements	Sequence (%)
Retroelements	2,940,839	42.54
SINEs:	1,524,273	12.99
Penelope:	668	0
LINEs:	933,206	21.01
L2/CR1/Rex	358,452	3.71
R2/R4/NeSL	412	0
RTE/Bov-B	13,040	0.13
L1/CIN4	561,061	17.16
LTR_elements	483,360	8.54
Gypsy/DIRS1	20,356	0.16
Retroviral	452,769	8.29
DNA_transposons	404,233	3.64
hobo-Activator	276,698	2.12
Tc1-IS630-Pogo	115,053	1.42
MULE-MuDR	2,036	0.03
PiggyBac	1,957	0.02
Tourist/Harbinger	364	0
Rolling-circles	1,562	0.01
Unclassified:	48,379	0.2
Total interspersed repeats		46.39

### DNA methylation

Global methylation assessment detected a mean of 80% methylation and 1.56% hydroxymethylation of the CpG sites for both muriqui samples, in line with methylation values from blood in other primates (Faulk 2023).

### Mitochondrial DNA

**Fig. 2.**
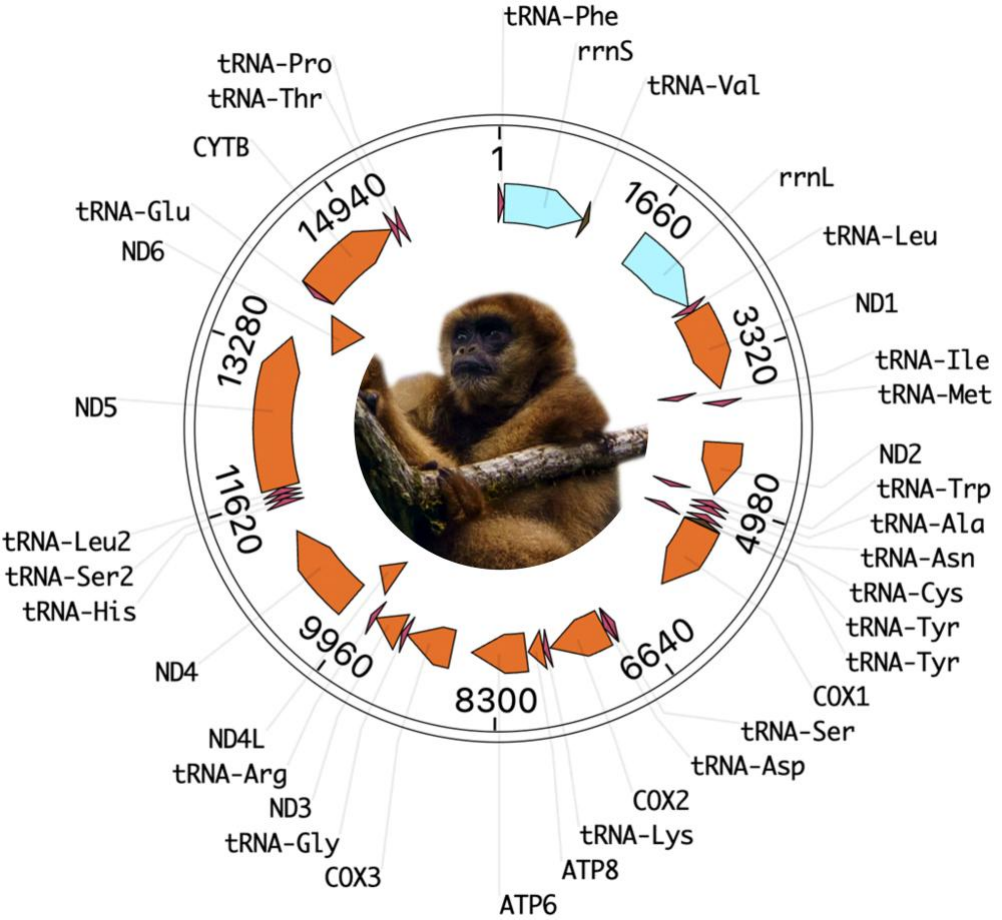
Mitogenome of the southern muriqui (*B. arachnoides*).

The mitogenome of the southern muriqui, Monalisa, was assembled from 11,000× coverage and consists of 16,562 bp over 37 genes, in line with the pattern of mammalians (Fig. 2). Serena's mitogenome was independently assembled to 15,564 bp and shared 99.47% sequencing identity with Monalisa. The nearest match in NCBI's database was equally distant to the *Lagothrix lagotricha* and *Lagothrix poeppigii* mitogenomes at 88.62% identity over 97% of their length. This result is in line with previous analyses of the mitochondrial DNA (mtDNA) in Atelids, which estimated that *Brachyteles* and *Lagothrix* diverged ∼8.5 million years ago (Di Fiore *et al.* 2015). Other closely related species with >85% identity were *Ateles chamek, Ateles marginatus, Ateles belzebuth, Ateles geoffroyi, Ateles paniscus, Alouatta caraya, Alouatta seniculus, Alouatta juara, Allouata palliata, Alouatta guariba, Alouatta macconnelli,* and *Alouatta discolor*, in descending order of sequence identity.

### Relevance to conservation

The southern muriqui population in situ is estimated to be <1,200 individuals across 20 subpopulations (Strier *et al.* 2017), but several of these subpopulations are small and restricted to isolated areas, which reduces their long-term viability. A recent study indicated that southern muriquis are predicted to lose between 28 and 35% of their climatically suitable habitat by 2050 due to climate change, and the combined effects of climate change and landscape fragmentation could reduce their suitable habitat to only 21% of their distribution range (Pinto *et al.* 2023; Pompeu and de Oliveira Portella 2023).

Reference genomes can help design strategies to improve the conservation status of a species. For example, we can identify traits relevant for local adaptations, which can inform conservation unit definitions and translocations. Additionally, we can develop a range of genetic tools such as microsatellite markers, single-nucleotide polymorphism panels, or reduced representation sequencing to investigate population genetics, which is important for predicting the long-term survival of a population (Paez *et al.* 2022).

Previous studies on muriqui genomics include sequencing of the southern muriqui transcriptome to investigate genes related to the immune system (Moreira *et al.* 2020) and sequencing of the mtDNA in both northern and southern muriquis to investigate phylogenetics (Chaves *et al.* 2019). Analyses of the mtDNA have also been used to assess population structure of northern muriquis (Fagundes *et al.* 2008), as well as their genetic diversity and historical events (Chaves *et al.* 2011).

Compared with nuclear DNA, mtDNA is shorter and has a higher mutation rate, which makes its sequencing more affordable and more effective in detecting evolutionary changes (reviewed by DeSalle *et al.* 2017). However, the mtDNA is restricted to maternal inheritance, precluding its use in determining paternal lineage, and it may cause taxonomic confusion due to potential introgression (Hurst and Jiggins 2005; Toews and Brelsford 2012; Seixas *et al.* 2018). The availability of a reference genome combined with the mitogenome can therefore be used to identify patterns of hybridization between sympatric species. Moreover, concerns about the accuracy of mtDNA in estimating demographic parameters pose limits to its use in population management (Cronin 1993; Moritz 1994). In contrast, nuclear genome is inherited by both parents and therefore provides a complete genetic make-up of an individual. Studies using genome-wide data have been important in wildlife conservation to protect important phenotypic traits associated with their survival, to identify genes which may be target of future analyses, and to make decisions for conservation priorities and strategies (Harrisson *et al.* 2014; Mable 2019; Hohenlohe *et al.* 2021; Theissinger *et al.* 2023). Therefore, the combination of the nuclear genome and the mitogenome can provide unique information to advance studies on muriqui conservation and evolution.

The genome assembled in our study is arguably the highest quality genome assembly among Atelids. A high-quality genome, which is more complete, contiguous, and correct, can increase the accuracy of structural variant analysis, runs of homozygosity, and comparative genomic and evolutionary analyses, even if it is not completely error-free (Brandies *et al.* 2019). This study illustrates the importance of zoological facilities in contributing to biodiversity conservation, and we strongly suggest that further efforts are needed to improve genomes within the Atelidae family. For this reason, we recommend that zoological and research facilities should, whenever possible, maintain a biological bank to store opportunistically acquired samples from endangered species. The methods described here can be used in-country to improve existing genomes and enhance studies focused on evolution and conservation of endangered species.

## Conclusion

We provide the first reference genomes for the southern muriqui using long reads through ONT’ PromethION. The quality of our best assembly was superior to reference genomes available for other closely related primate species, with a 99.5% BUSCO score, 319 contigs, an N50 of 58.8 Mb, and an L50 of 17. In addition, we reported the mitogenome and the global methylation patterns for this species. The availability of a reference genome for the southern muriqui is useful for studies on population genetics and will contribute to conservation efforts for this critically endangered species.

## Supplementary Material

jkaf034_Supplementary_Data

## Data Availability

This genome assembly and annotation have been deposited at NCBI accession numbers JBLDXS000000000 and JBLDYO000000000, under project numbers PRJNA1187200, PRJNA1187189, PRJNA1187204, and PRJNA1187201 and BioSamples SAMN44786511 and SAMN44786512. The script for the assembly is detailed in Supplementary File 1. The protein annotation was saved in GFF (Supplementary File 2). Both files are available as Supplemental materials. Supplemental material available at G3 online.
