# Supplementary material for: De novo whole-genome assembly of the critically endangered southern muriqui (Brachyteles arachnoides)
Source: G3 (Bethesda). 2025 Feb 17;15(4):jkaf034. doi: 10.1093/g3journal/jkaf034 (PMC12005144; doi:10.1093/g3journal/jkaf034)
Supplement: jkaf034_Supplementary_Data [file jkaf034_supplementary_data.zip › jkaf034_Supplementary_Data.pdf]

Supplementary Material

De novo whole genome assembly of the critically endangered Southern muriqui (*Brachyteles arachnoides*) by Faulk et al.

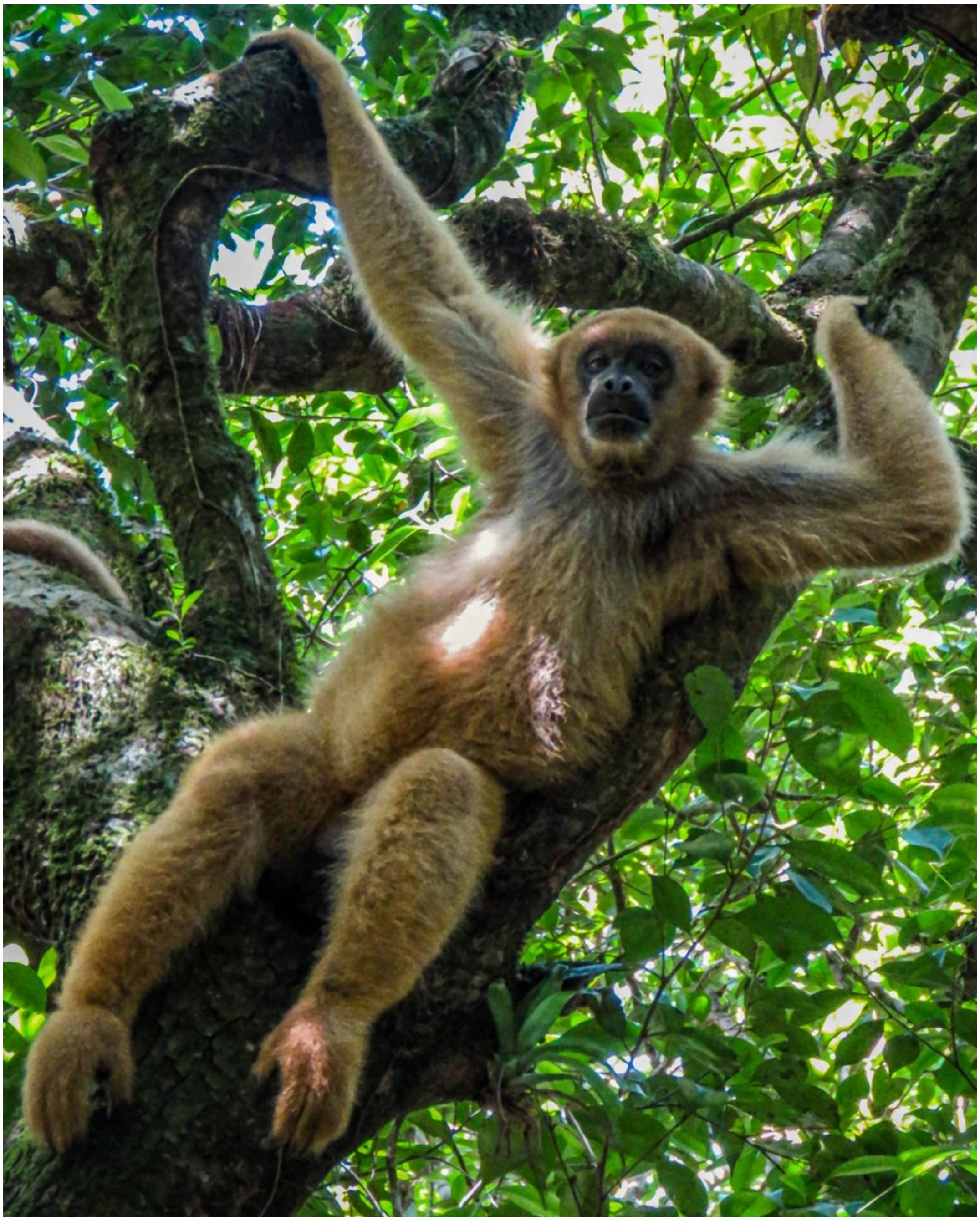

Table of Contents

Project 2
Basecalling 2
Read QC and filtering 3
Correcting Reads 3
Assembly hifiasm 3
Assembly flye 4
Foreign Contaminant Screen 4
Purge_dups 5
Scaffolding 5
QC and BUSCO 6
MitoHiFi 6
Mitochondrial phylogeny 6
Repeats 6
RepeatModeler 6
RepeatMasker 7
Align reads to final genomes 7
Diploid with HapDup 7
Methylation 8
GeMoMa Gene Annotation 8

Project

Sequencing of samples from Brazil for genome assembly. Samples were sequenced on the P2 Solo on R10.4.1 flowcells.

Basecalling

Basecalling was performed in minknow during sequencing using the dna_r10.4.1_e8.2_400bps_sup@v4.3.0 model. Re-basecalled with dna_r10.4.1_e8.2_400bps_sup@v5.0.0.

```
# Posthoc basecalling
# Model dna_r10.4.1_e8.2_400bps_sup@v5.0.0
~/Desktop/dorado-0.7.3-linux-x64/bin/dorado basecaller sup,5mCG_5hmCG
<input_directory> -r --min-qscore 10 > <output_file>.mod.bam
```

```
# Demux barcoded bams
~/Desktop/dorado-0.7.3-linux-x64/bin/dorado demux --output-dir
Muriqui_barcodes_7-24-24_demux --kit-name SQK-NBD114-96 Muriqui_barcodes_7-
24-24.mod.bam
```

Read QC and filtering

```
# Filtered for reads with quality score > 10
samtools view -b -e '[qs]>=10' BloodofChildren_4433_6-17-
24.d0.7.2.5.5mCG_5hmCG.hg38.modmapped.bam > BloodofChildren_4433_6-17-
24.d0.7.2.5.5mCG_5hmCG.hg38.modmapped.q10.bam

# Stats on read quality
samtools fastq file.bam | seqkit stats -a -
```

Correcting Reads

```
# Run Dorado correct to correct reads > 5kb prior to assembly.
dorado correct file.fastq > file.corr.fasta

# On MSI
(base) cfaulk@ahl04 [/home/faulkc/cfaulk/brazil_processing] % cat
dorado_correct_serena.sh
#!/bin/bash, eval=FALSE -l
#SBATCH -A faulkc
#SBATCH --time=24:00:00
#SBATCH -p msigpu
#SBATCH --ntasks=128
#SBATCH --mem=495g
#SBATCH --mail-type=ALL
#SBATCH --mail-user=cfaulk@umn.edu

cd /home/faulkc/cfaulk/brazil_processing

./dorado-0.7.3-linux-x64/bin/dorado correct Muriqui_Serena.fastq >
Muriqui_Serena.corr.fasta
```

Assembly hifiasm

```
# Use hifiasm
# Process resulting asm file to fasta
awk '/^S/{print ">"$2;print $3}' Muriqui_Monalisa.asm.bp.hap1.p_ctg.gfa >
Muriqui_Monalisa.asm.bp.hap1.p_ctg.fa

# Slurm job hifiasm
(base) cfaulk@ahl01 [~/brazil_processing] % cat Muriqui_Monalisa_hifiasm.sh
#!/bin/bash, eval=FALSE -l
#SBATCH -A faulkc
#SBATCH --time=24:00:00
#SBATCH -p msibigmem
```

```
#SBATCH --ntasks=128
#SBATCH --mem=1995g
#SBATCH --mail-type=ALL
#SBATCH --mail-user=cfaulk@umn.edu

cd /home/faulkc/cfaulk/brazil_processing/
/home/faulkc/cfaulk/hifiasm/hifiasm -o Muriqui_Monalisa.asm -t 128
Muriqui_Monalisa_7-22-24.corr.fasta
```

Assembly flye

```
# Use flye 2.9.5

# Slurm file
(base) cfaulk@ahl04 [/home/faulkc/cfaulk/brazil_processing] % cat
flye_monalisa.raw.sh
#!/bin/bash, eval=FALSE -l
#SBATCH -A faulkc
#SBATCH --time=24:00:00
#SBATCH -p ag2tb
#SBATCH --ntasks=128
#SBATCH --mem=1995g
#SBATCH --mail-type=ALL
#SBATCH --mail-user=cfaulk@umn.edu

cd /home/faulkc/cfaulk/brazil_processing
#conda activate flye

flye --nano-raw Muriqui_Monalisa_7-22-24.corr.fasta.gz --out-dir
muriqui_monalisa_flye-raw --threads 128
```

Foreign Contaminant Screen

```
# Required prior to NIH ubmission.
# FCS-adapt removes adapter and vector sequences

# Install
curl -LO https://github.com/ncbi/fcs/raw/main/dist/run_fcsadaptor.sh
chmod 755 run_fcsadaptor.sh
curl https://ftp.ncbi.nlm.nih.gov/genomes/TOOLS/FCS/releases/latest/fcs-
adaptor.sif -Lo fcs-adaptor.sif

# Run
sudo ./run_fcsadaptor.sh --fasta-input assembly.fasta --output-dir . --euk --
container-engine singularity --image fcs-adaptor.sif

# Clean the genome (26 bp dropped)
curl -LO https://github.com/ncbi/fcs/raw/main/dist/fcs.py
cat assembly.fasta | sudo python3 fcs.py clean genome --action-report
./fcs_adaptor_report.txt --output clean.fasta --contam-fasta-out contam.fasta
```

Purge_dups

Purge_dups was run to remove haplotigs and contig overlaps in a de novo assembly based on read depth.

```
# Install
git clone https://github.com/dfguan/purge_dups.git
cd purge_dups/src && make -j 16

# Align the data to generate paf files
# minimap2 -x map-ont consensus.fasta $i | gzip -c - > $i.paf.gz
minimap2 -I 4G -x map-ont -t 32 clean.fasta ../../Muriqui_Monalisa_7-22-
24.mod.bam.fastq.gz | pigz > muriqui_monalisa.paf.gz

# Produce stats and cutoffs file
./purge_dups/bin/pbcstat muriqui_monalisa.paf.gz
./purge_dups/bin/calcuts PB.stat > cutoffs 2>calcults.log

# Split consensus and self-align
./purge_dups/bin/split_fa clean.fasta > clean.split.fa
minimap2 -xasm5 -DP clean.split.fa clean.split.fa | gzip -c - > muriqui-
monalisa.split.self.paf.gz

# Purge dups and haplotigs
./purge_dups/bin/purge_dups -2 -T cutoffs -c PB.base.cov muriqui-
monalisa.split.self.paf.gz > dups.bed 2> purge_dups.log

# Get purged primary and haplotigs
./purge_dups/bin/get_seqs -e dups.bed clean.fasta

# Generate histogram
./purge_dups/scripts/hist_plot.py -c cutoffs PB.stat PB.base.png
```

Scaffolding

```
# Install ntLink for scaffolding with gap-filling
mamba install -c bioconda -c conda-forge ntlink

# Run with 3 rounds
mkdir ntlink-purged
cd ntlink-purged
ln -s ../purged.fa
ln -s ../../../Muriqui_Monalisa_7-22-24.mod.bam.fastq.gz
ntLink_rounds run_rounds_gaps target=purged.fa reads=Muriqui_Monalisa_7-22-
24.mod.bam.fastq.gz k=32 w=100 t=5 rounds=3

# Split sequences by N to remove unfilled gaps
awk '/^>/ {print $0; next} {gsub(/[nN]+/, "\n>contig_subseq" ++i "\n");
print}' purged.fa.k32.w100.z1000.ntLink.3rounds.fa > purged-linked-nogaps.fa
```

QC and BUSCO

```
# Read stats
# Assembly-stats
https://github.com/sanger-pathogens/assembly-stats

# Compleasm
conda create -n compleasm -c conda-forge -c bioconda compleasm
conda activate compleasm
compleasm run -t 32 -l primates -L ~/Desktop/genomes/mb_downloads -a
assembly.fasta -o compleasm-purged-muriqui_monalisa
```

MitoHiFi

```
# Pull docker container
sudo docker pull ghcr.io/marcelauliano/mitohifi:master

# Run through singularity
singularity shell --bind /home/carrie/Desktop/brazil_processing/final-
assemblies/MitoHiFi/:/MitoHiFi docker://ghcr.io/marcelauliano/mitohifi:master
mitohifi.py -h
cd MitoHiFi
Singularity> findMitoReference.py --species "Ateles geoffroyi" --outfolder .
--min_length 14000
Singularity> mitohifi.py -r Muriqui_Monalisa_7-22-24.mod.bam.fasta -f
NC_019800.1.fasta -g NC_019800.1.gb -t 32 -o 2

# The "potential contigs" directory lists the contig containing mtDNA
(contig_12841).
# Annotations were saved for submission to NCBI Assembly.
```

Mitochondrial phylogeny

```
# Install MAFFT and iqtree2

# Align
mafft --auto muriqui_mitogenomes.fa > muriqui_mitogenomes.aln

# Tree
iqtree2 -s muriqui_mitogenomes.aln

# Visualize with FigTree
```

Repeats

RepeatModeler

```
# Install Singularity
wget
https://github.com/sylabs/singularity/releases/download/v4.1.4/singularity-
```

```
ce_4.1.4-jammy_amd64.deb

# Install DFAM TEtools
curl -sSLO https://github.com/Dfam-consortium/TETools/raw/master/dfam-
tetools.sh
chmod +x dfam-tetools.sh
./dfam-tetools.sh

# Build database
BuildDatabase -name rm-monalisa final_monalisa_plus_mt.fa

# Run RepeatModeler
RepeatModeler -database rm-monalisa -LTRStruct -threads 32
```

RepeatMasker

```
# Default library from DFAM
RepeatMasker -spec primates -s -pa 32 -xsmall -gff -e rmblast
final_monalisa_plus_mt.fa

# Custom library created with RepeatModeler2
RepeatMasker -lib rm-monalisa-families.fa -s -pa 32 -xsmall -gff -e rmblast
final_monalisa_plus_mt.fa
```

Align reads to final genomes

```
# Create modmapped bam
~/Desktop/dorado-0.8.0-linux-x64/bin/dorado aligner final-
assemblies/final_monalisa_plus_mt.fa Muriqui_Monalisa_7-22-24.mod.bam >
Muriqui_Monalisa_7-22-24.modmapped.bam
```

Diploid with HapDup

There is no option to create a diploid genome with flye so HapDup is recommended to recover the other haplotype.

```
# SKIPPED Map the reads to the assembly
#minimap2 -ax map-ont -t 30 assembly.fasta reads.fastq | samtools sort -@ 4 -
m 4G > lr_mapping.bam
#samtools index -@ 4 assembly_lr_mapping.bam

# Run with singularity
singularity pull docker://mkolmogo/hapdup:0.12
HD_DIR=`pwd`
singularity exec --bind $HD_DIR hapdup_0.12.sif hapdup --assembly
$HD_DIR/final_monalisa_plus_mt.fa --bam $HD_DIR/Muriqui_Monalisa_7-22-
24.modmapped.bam --out-dir $HD_DIR/hapdup -t 32 --rtype ont

# Output
hapdup_dual_{1,2}.fasta - dual assembly
```

```
phased_blocks_hp{1,2}.bed - phased blocks coordinates (in dual assmeblies)
hapdup_phased_{1,2}.fasta - haplotype-resolved assmebly
```

Methylation

```
# Modkit
~/Desktop/modkit_0.4.1/modkit pileup --ref ../final-
assemblies/final_monalisa_plus_mt.fa --cpg ../Muriqui_Monalisa_7-22-
24.modmapped.bam Muriqui_Monalisa_7-22-24.modmapped.bam.bed

# Summarize
for i in *.bed; do  awk -v file="$i" '$4=="m" {can+=$13; mod+=$12; oth+=$14;
valid+=$10} END{print file "\tCpG canonical " (can/valid) "\tCpG methyl "
(mod/valid) "\tCpG hydroxy " (oth/valid)}' $i >> methylation-summary.txt;
done
```

GeMoMa Gene Annotation

Wiki

```
# Install dependency `mmseq`:
mamba install -c conda-forge -c bioconda mmseqs2

Installed version 1.8 [@Keilwagen:2018:GeMoMa_RNAseq; @Keilwagen:2016:GeMoMa]
by downloading `.zip` file from
[website](https://www.jstacs.de/index.php/GeMoMa#Requirements)

# Run vs Human annotations
#The human T2T genome with annotations was used as a reference.

java -Xmx50g -jar GeMoMa/GeMoMa-1.9.jar CLI GeMoMaPipeline threads=16
outdir=annotation GeMoMa.Score=ReAlign AnnotationFinalizer.r=NO o=true
t=../final-assemblies/muriqui_monalisa_hapdup_dual_1.fasta.gz i=H_sapiens
a=GCF_000001405.40_GRCh38.p14_genomic.gff.gz
g=GCF_000001405.40_GRCh38.p14_genomic.fna.gz`

# Busco on protein mode:
compleasm download primates
compleasm protein -p predicted_proteins.fasta -l primates -o compleasm-
proteins -t 32
```
